# Supplementary material for: Pure Acetylene Semihydrogenation over Ni–Cu Bimetallic Catalysts: Effect of the Cu/Ni Ratio on Catalytic Performance
Source: Nanomaterials (Basel). 2020 Mar 11;10(3):509. doi: 10.3390/nano10030509 (PMC7153591; doi:10.3390/nano10030509)
Supplement: Supplementary file 1 [file nanomaterials-10-00509-s001.pdf]

Supplementary Information

Pure acetylene semihydrogenation over Ni–Cu bimetallic catalysts: Effect of the Cu/Ni ratio on catalytic performance

[1] College of Chemistry and Chemical Engineering of Yantai University, Yantai, Shandong 264004, P. R. China, lhkang@ytu.edu.cn (L.K.); zhuminyuan@shzu.edu (M.Z.)

[2] School of Chemistry and Chemical Engineering of Shihezi University, Shihezi, Xinjiang 832003, P. R. China, Shuzhenzhou@163.com (S.Z.); lhkang@ytu.edu.cn (L.K.); zhouxuening@stu.shzu.edu.cn (X.Z.); xz20200217@126.com (Z.X.)

* Correspondence: zhuminyuan@shzu.edu.cn; Tel.: +86- 993-205-7270

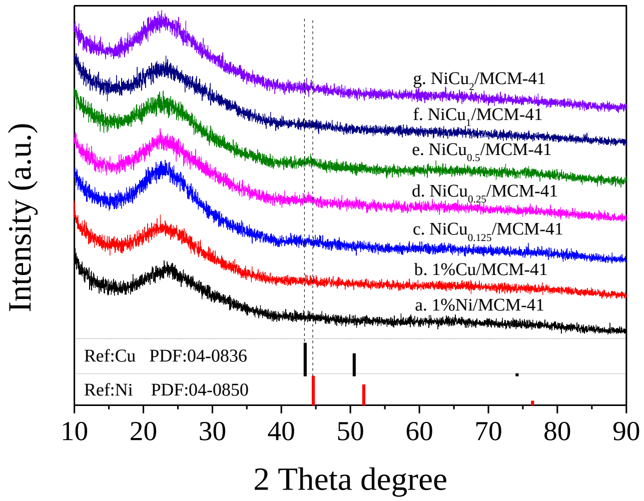

Figure S1. XRD patterns of the catalysts.

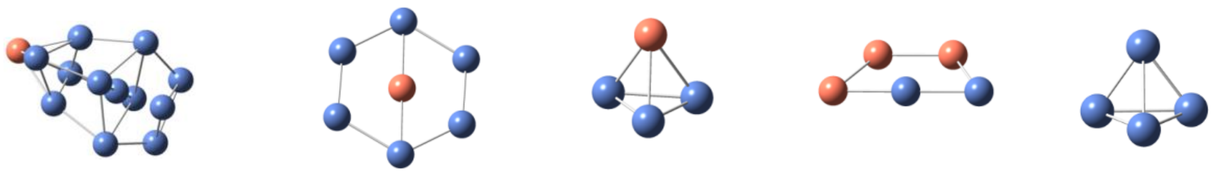

$NiCu_{0.125}$ **$NiCu_{0.25}$** **$NiCu_{0.5}$** **$NiCu_2$** **Ni**

Figure S2. Optimal structure of the catalysts. Nickel and copper atoms are depicted in blue and red, respectively.

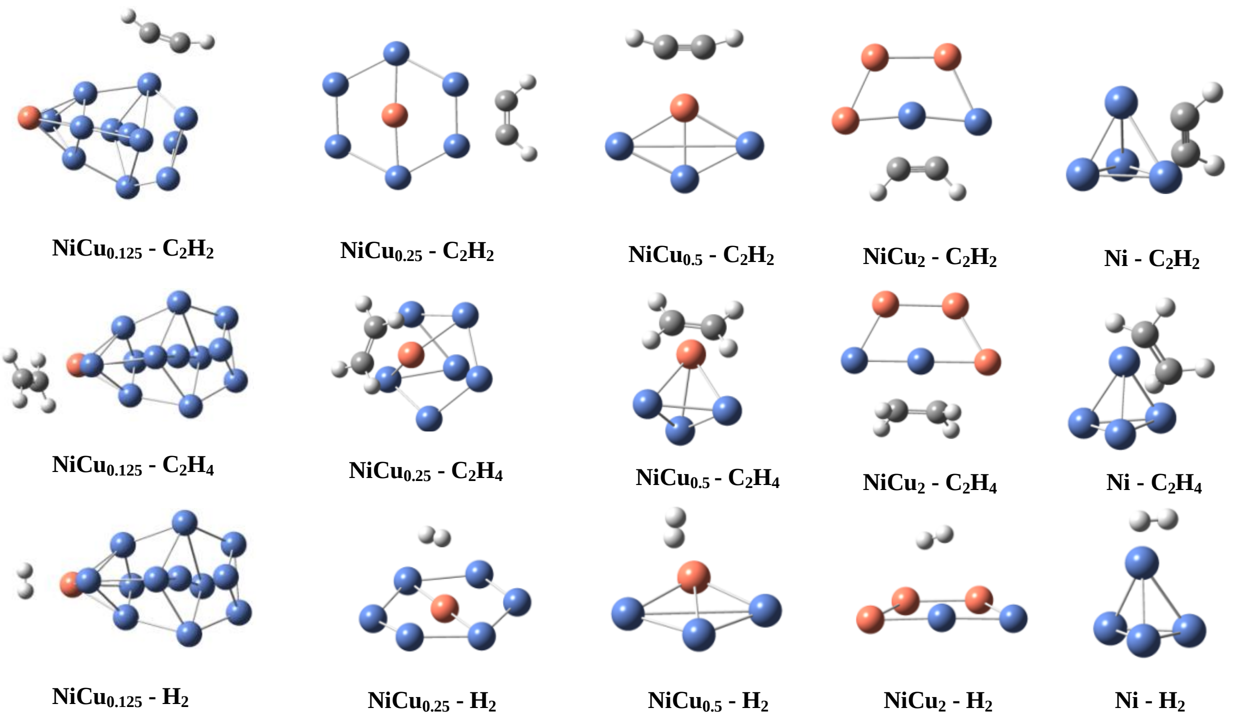

Figure S3. Adsorption configuration of C_2H_2, C_2H_4, and H_2 on several catalysts. Carbon, hydrogen, nickel, and copper atoms are depicted in gray, white, blue, and red, respectively.

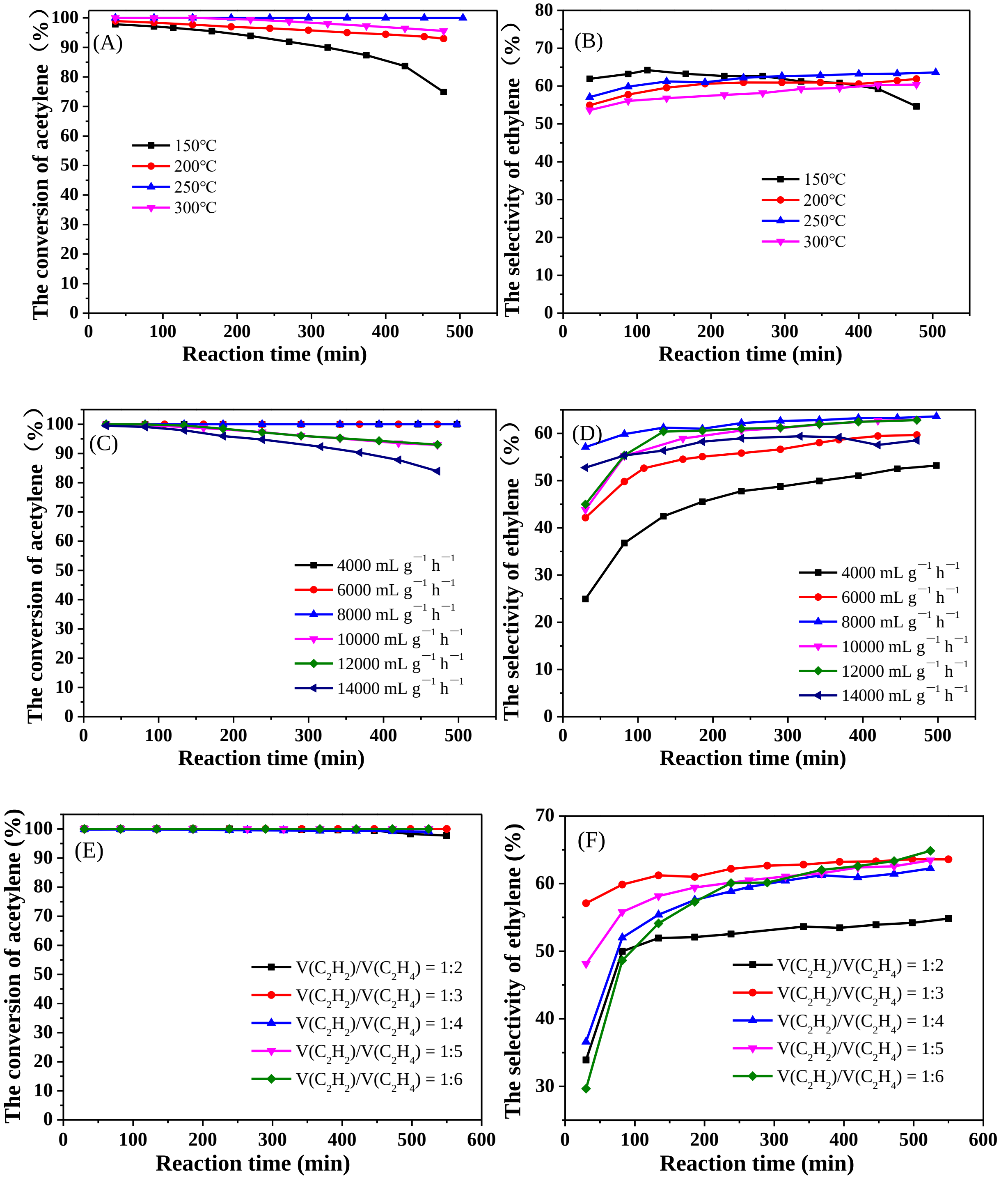

Figure S4. Effect of reaction temperature on (A) acetylene conversion and (B) ethylene selectivity at 8000 mL mg^{-1} h^{-1} and $V(H_2)/V(C_2H_2)$ = 3. Effect of acetylene space velocity on (C) acetylene conversion and (D) ethylene selectivity at 250 °C and $V(H_2)/V(C_2H_2)$ = 3. Effect of acetylene to hydrogen ratio on (E) acetylene conversion and (F) ethylene selectivity at 250 °C and 8000 mL mg^{-1} h^{-1}.
